# Cat Mammary Tumors: Genetic Models for the Human Counterpart

**DOI:** 10.3390/vetsci3030017

**Published:** 2016-08-16

**Authors:** Filomena Adega, Ana Borges, Raquel Chaves

**Affiliations:** 1 Laboratory of Cytogenomics and Animal Genomics (CAG), Department of Genetics and Biotechnology (DGB), University of Trás-os-Montes and Alto Douro (UTAD), Vila Real 5001-801, Portugal; filadega@utad.pt (F.A.); borgesluisa@sapo.pt (A.B.); 2 Faculty of Sciences, BioISI—Biosystems & Integrative Sciences Institute, University of Lisboa, Campo Grande, Lisboa 1749-016, Portugal

**Keywords:** feline mammary carcinomas, cancer critical genes, chromosome rearrangements, cell and animal model, targeting therapies

## Abstract

The records are not clear, but Man has been sheltering the cat inside his home for over 12,000 years. The close proximity of this companion animal, however, goes beyond sharing the same roof; it extends to the great similarity found at the cellular and molecular levels. Researchers have found a striking resemblance between subtypes of feline mammary tumors and their human counterparts that goes from the genes to the pathways involved in cancer initiation and progression. Spontaneous cat mammary pre-invasive intraepithelial lesions (hyperplasias and neoplasias) and malignant lesions seem to share a wide repertoire of molecular features with their human counterparts. In the present review, we tried to compile all the genetics aspects published (i.e., chromosomal alterations, critical cancer genes and their expression) regarding cat mammary tumors, which support the cat as a valuable alternative in vitro cell and animal model (i.e., cat mammary cell lines and the spontaneous tumors, respectively), but also to present a critical point of view of some of the issues that really need to be investigated in future research.

## 1. Introduction

The occurrence of spontaneous tumors in some animals makes them suitable models for the study of tumorigenesis, for the improvement of diagnostic tools or for the development of successful therapies for the human counterpart. Moreover, the shorter lifespan and faster progression of cancer in model species allows a quicker trial completion and data collection [1]. Amongst the animal models used in comparative oncology, cats (*Felis catus*) are emerging as an excellent organism, a conclusion supported by different perspectives. Unlike the usual laboratory animals, such as rodents, cats (and dogs) share the same environmental risk factors as humans, with cancer occurring naturally in this species, contrarily to rodents (frequently immunocompromised). Moreover, comparative genomics has revealed that there is a larger homology between cats and humans for key cancer-related genes (e.g., [2,3,4]) than there is between humans and other animal models [5,6]. In fact, cats, as companion animals, share similar environmental risk factors as humans, what is, most probably reflected in the tumor biology, allowing to disclose the complex interplay of the environment with the genome. The various spontaneously occurring cancers in cats represent an exceptional opportunity for translational advances and for both the human and the feline healthcare, as similar biology can bring mutual benefit [7].

Feline mammary carcinomas (FMCs) are the third most common type of neoplasm in cats and a large part of these (90%) are malignant [8,9]. It seems that there is an extremely decreased risk of feline mammary tumors development in cats spayed before 6 months and 1 year of age and, inversely, an increased risk in cats under regular, continued progestin treatment. Nevertheless, the biological behavior of FMTs in intact and in spayed cats seems to be identical (reviewed by Zappulli and colleagues [10]). The malignant tumor types in cats are most frequently carcinomas, particularly simple carcinomas as tubolopapillary and solid carcinomas (e.g., [10]). FMCs are usually highly aggressive, infiltrative and metastatic, displaying a great uncertainty in the outcome, not readily predicted at the time of diagnosis [11]. The feline mammary carcinoma shares a broad clinicopathologic, demographic and epidemiological similarity with the human breast carcinoma (HBC) [12]. In accordance with the criterion of the WHO classification of mammary tumors, human breast carcinomas are simple glandular epithelial tumors. In this sense, FMCs display a higher resemblance to their human counterparts than to those of dogs, since in cats the vast majority of FMCs are malignant glandular epithelial tumors, and in dogs only about 45% of mammary tumors are malignant and display a much higher number of complex and mixed tumors than cats and humans (e.g., [13,14,15]). 

Although there is evidence pointing to steroid hormone involvement in FMCs, the vast majority of them are estrogen, progesterone and HER2 receptors negative (triple negative) [16,17,18], constituting a remarkable spontaneous model for the human triple negative breast cancers. These are extremely aggressive, not responding to hormone-antagonists therapies, with a poor prognosis [9]. The lack of effective targeted therapies boosts the search for models that efficiently mimic the human biological system. Comparative genomics and specifically, comparative oncogenomics can hold the tools to choose the more appropriate model organism for a determined cancer type or subtype on the basis of sharing similar patterns, going from the basic DNA sequence, to its expression in mRNA, to protein formation, functional status, amount or subcellular location. 

## 2. “Cytogenomics” of Cat Mammary Tumors

Despite the emerging high-resolution molecular technologies for genome analysis, the examination of a cell’s, a tissue’s or an organism’s karyotype still is the simplest, cheapest and fastest way of observing in one glimpse its whole genome. Cytogenetics thus provides a global view of the organization of any genome. 

Tumor cells are characterized by a great genome instability that can be frequently observed at the chromosome level. In humans, the cytogenetic analysis of tumors is more than a genome analysis, it is an important diagnosis tool for cancer-specific chromosome alterations using those as prognostic or predictive biomarkers, being in the latter case indicative of the likelihood of benefiting from a specific therapy. This relies on the fact that particular chromosome alterations are recurrently found in specific cancers (e.g., [19]). In terms of cytogenetic analysis of feline mammary carcinomas, the first published studies were performed by Minke et al. [20] in two established cat mammary cell lines (same origin) using conventional chromosome banding. These authors identified loss of several chromosomes, namely, A3, D2, F1 and F2 in one of the cell lines (named K248C); and losses of chromosomes B4, F1 and F2 and gain of chromosome C2 in the other cell line (named K248P). Additionally, in other works, several marker chromosomes were detected whose identification was difficult due, most probably, to the technique’s resolution [21,22,23]. Some of these seemed to involve chromosomes B1, B2 and D4. Later, Mayr et al. [22] reported recurrent losses of chromosome B2-material and E3-material in cat mammary gland neoplasms in cells exhibiting highly reshuffled karyotypes. More recently, Borges et al. [24] identified several different aneuploidies in different passages of a feline mammary cancer cell line (FkMTp). Although the modal value was 38 (the standard chromosome number of the species) or near 38 for the majority of the passages, some few passages displayed a wide variation in the chromosome number (2n = 26–252). Preliminary data of this cell line revealed a high degree of genome instability manifested in a form of numerous chromosome rearrangements involving different chromosomes (unpublished work). Interestingly, some of these chromosomes are the same as the ones involved in previous cytogenetic analysis performed by Minke et al. [20] and Mayr et al. [21,22], pointing to a recurrent use of these in the tumorigenesis of cat mammary tumors. Moreover, some of these chromosomes are syntenic to human chromosome regions already reported as being associated with human breast cancer (e.g., [25,26,27,28,29]). In most of these HBC chromosomes, the observed genomic imbalances were determined via comparative genomic hybridization (CGH), a technology now being applied also in the analysis of cat tumors, but for now only reported in feline sarcomas [30]. 

The gross conservation of the human and domestic cat (*Felis catus*, FCA) karyotypes has been documented from the first comparative chromosome banding patterns performed by Nash and O’Brien in 1982 [31]. Since then, molecular cytogenetics [32,33,34] and genome sequencing ([35,36,37,38], Ensembl Genome browser database) have reinforced these findings, indicating a high degree of conservation between the human and the domestic cat genomes, supposed to have diverged more than 92 million years ago from their common ancestor [9]. This great homology between the cat and the human genome allows to perform valuable inferences/extrapolations from the cat to the human counterpart at the level of the chromosomes, the DNA, the RNA and the proteins presumably involved in specific tumorigenic processes/events. 

## 3. Cat Critical Cancer Genes: A Sequence Perspective

The pathogenesis and progression of breast cancer have been connected to a number of critical genes, those that control cell division and growth, apoptosis or the ones that repair damaged DNA [39,40,41]. The somatic alterations in the genome that result in tumor initiation can be viewed as a disequilibrium in a scale where oncogenes are activated by mutation or copy number gain, and tumor suppressor genes are inactivated by mutation or copy number loss, culminating in an antagonist scenario to that of a normal genome [42,43,44]. Besides the copy number variations of these opposing genes, point mutations are known to be also important triggers of the deleterious gains and losses of function observed in oncogenes and tumor suppressor genes, respectively, in a variety of cancers [45,46]. It is believed that acquired somatic mutations account for approximately 90% of human breast tumors [47]. 

The similarity and homology for key cancer-related genes is far superior between humans and cats than between rodents (e.g., [6]). From the genes probably involved in cat mammary tumors, some are viewed as critical (highly important for the tumorigenic process) and for that reason great importance has been attributed to them (Table 1). Amongst these, *TP53* suppressor gene and growth factor genes, such as the epithelial growth factor receptor family (*EGFR* family, mainly the erbB-2 Receptor Tyrosine Kinase 2, usually named *ERBB2*, *EGFR2*, *HER2* or *NEU*) (e.g., [4,39,40,41]), have been the most studied at the sequence level. Some other genes have attracted the attention of researchers, but at a lower level. Examples are the Macrophage Stimulating 1 Receptor (*MST1R*, *RON*, or *STK*) [48], the *TWIST1* oncogene [49], the mRNA of estrogen receptor gene (*ER*) [50], or *CyclinA* [51] (Table 1). Some other human breast cancer key genes were also analyzed in feline mammary carcinomas at the sequence level (e.g., the tumor suppressor and cell cycle regulatory genes *p21 WAF1* and *p27 Kip1* by Mayr et al. in 2000 [52] or the oncogene *RAS*, specifically *N-*, *Ki-*, and *Ha-ras* performed by Watzinger et al. [53]), but the search for any mutational signal in the latter referred genes failed. Although these results are apparently negative [53] from the classical gene activation point of view, the fact that they do not present an identifiable mutation (either copy number variations or point mutations) do not imply that they do not play a role in the pathogenesis or progression of feline mammary tumors.

Mutations in the *TP53* suppressor gene still represents one of the most frequently detected genetic alterations in human neoplasms (e.g., [54,55,56,57,58]). Though less mutated in breast cancer than in other tumors, *TP53* missense mutations seem to be among the key driving factors in triple negative breast cancer, the most aggressive human breast cancer subgroup known [59]. It seems that it results from the crosstalk between the mutant p53 protein and other oncogenic pathways in breast cancer [59]. In cats, *TP53* mutations have been reported in various cat tumors, including mammary carcinomas [60,61,62]. Mayr et al. [61,62] found a missense mutation in codon 282 and a 9 bp deletion (ATC.ATC.ACC) in codons 254–256 in exon 7. Later, Mayr et al. [60] analyzed the region from exon 4 to 8 and identified missense mutations in the exon 5 in other feline mammary carcinomas. Recent studies on *TP53* suggest that there are still unknown functional intersections of the mutant p53 with cellular pathways. However, the definition and validation of these suppositions should be performed in breast cancer models [59], such as feline mammary tumors, which share the same type of DNA mutations in this cancer key player. 

Regarding the DNA sequence of the growth factor erbB-2 receptor tyrosine kinase 2 gene (*ERBB2*), this has been investigated for the first time in cat by Santos and collaborators in 2012 [4] and 2013 [41]. Also in 2013, Soares et al. [63] analyzed this gene by in situ hybridization to verify the number of copies in 10 feline mammary carcinomas samples and found no gene amplification. Santos and collaborators [4] identified two sequence variants and two haplotypes specific for the cat mammary tumors samples analyzed (exons 17–20). A probable association between the clinicopathological traits and the variant haplotypes was predicted. It seems that there is a direct association between the primary tumor size (one of the two most important prognostic factors) and the number of masses with the cat *ERBB2* variant haplotypes identified, revealing the significance of the analysis of this gene not only in the veterinary medicine, but also at the comparative oncology level [4]. Later, Santos and collaborators [41] extended the search for *ERBB2* gene sequence variants in cat mammary lesions (neoplastic and non-neoplastic) to the region encompassing exons 10–15, which encodes part of the extracellular domain of the erbB-2 protein. They found three amino acid changes, corresponding to three non-synonymous genomic variants specific for the cat mammary lesions analyzed. These authors performed a computational analysis and homology modelling and predicted that these variants can damage the 3D structure of the protein. These studies point to a prognostic value of the *ERBB2* gene in cat mammary tumors, and as a result, the use of this animal as model for the human equivalent. Additionally, and importantly, the finding of a homologous gene behavior and the recurrent occurrence of low erbB-2 expression levels in cat mammary tumors suggests that cat mammary neoplasias are a valuable model for erbB-2 negative human breast cancer [41]. 

Soares and colleagues [63] analyzed the status of the topoisomerase II alpha gene (*TOP2A*) in the FMCs referred above, as the status of these two genes, *ERBB2* and *TOP2A*, is usually “in accordance” in some human breast cancers [64,65]. These authors did not detect amplification in the *ERBB2* negative, as it happens in human ERRB2- breast cancer cases [63].

DeMaria et al. [48] studied the feline *MST1R* sequences and found a high level of similarity between the homologous human gene exons that encode the juxtamembrane and transmembrane domains of the *MST1R* receptors. This receptor belongs to the MET family and it is believed to be involved in the activation of the tyrosine kinase cascade providing invasive properties to carcinoma cells [66]. Point mutations in the *MST1R* kinase gene can activate the *MET* gene in human cancers releasing the oncogenic and metastatic potential of the receptor [67]. Feline *MST1R*-specific transcript was detected by reverse transcriptase PCR (RT-PCR) in seven of eight analyzed feline mammary carcinomas. These findings point *MST1R* as a potential critical gene involved in the invasiveness of feline tumors, as what is believed to happen in humans, and its mutational proneness must not be neglected. 

Cardazzo et al. [50] analyzed the sequence of the feline estrogen receptor 1 (*ESR1*) mRNAs. In human breast tissues or cell lines the isoform ER*_*E7 [68,69] is the more abundant, what is in accordance with the results of their work in cat mammary tumors, where this isoform was observed in all the analyzed samples (24 feline mammary carcinomas and 15 normal mammary glands). The second most frequently isoform in the cat is ER*_*E4, a variant that is also quite frequent in human normal and neoplastic breast tissues [68,69,70]. Furthermore, similarly to what takes place in human tissues, in addition to the wild-type ER-mRNA, several exon deleted splicing variants were also found in various feline tissues, in opposition to what is observed in rodents, for instance (in [50]). Once again, the results of this work support the use of cat as a good model for the analysis of this gene and its transcripts variants in breast cancer, in comparison with rodents that show considerable divergent data [50]. 

Some years ago, Sahlin et al. [71] suggested that germline mutations in the oncogene/proto-oncogene *TWIST1* may predispose to breast cancer. *TWIST1* encodes the Twist-1 protein, a basic helix-loop-helix DNA-binding transcription factor [72]. Baptista et al., in 2011 [49] searched for *TWIST1* coding region variations in 45 feline mammary gland lesions. Although these authors were not able to identify any mutation in this region, including the mutation described in human breast cancer (c.309C > G, [reviewed in 49]), they found two germline variants in the *TWIST1* gene intron in some of the carcinomas’ samples, namely GQ167299:g.535delG and GQ167299:g.460C > T [49]. Introns or intronic sequences’ retention in a mature mRNA is a form of alternative splicing resulting in multiple transcripts and protein variants that may culminate in different functions from the same gene [73]. The intronic mutations detected by Baptista et al. [49] should thus be taken into consideration, as these can be potential sources for expression variation of the *TWIST1* oncogene. 

The fact that around 95% of the human genes are alternatively spliced (e.g., [74]) is driving the investigation into its implications. Moreover, and very recently, Marquez et al. [75] proposed another form of alternative splicing he called exitron splicing, that is regulated across tissues in response to stress and in carcinogenesis. 

From the genes so far analyzed in cat mammary tumors, an obvious conclusion stands out: there is a clear resemblance of their sequence status with the human counterpart. Yet, the data gathered here can be viewed as an “in progress” catalogue of critical cancer genes that can be studied in the feline mammary tumor model. This continuing task still lacks a bulk of knowledge even in the genes already analyzed, including larger sampling, detailed molecular sequence variation profiling, transcripts’ isoforms identification and the possible implications those might have in the tumorigenesis process. We firmly believe that as more key-cancer genes are analyzed, the feline mammary tumor model will be used. Key to this achievement will be the full sequencing and assembly of the cat genome that it is still in an ongoing phase. Additionally, but at a much more complex level, the cat epigenome analysis will surely provide important information on the mechanisms regulating the sequences involved in cancer initiation, promotion and progression, allowing an even more reliable comparative oncogenomics.

## 4. Cat Critical Cancer Genes: From Gene Expression Patterns to Pathways

Notwithstanding the DNA sequence, the precise functions of a gene can only be ascertained by the analysis of its products, either in the form of transcripts (as regulatory RNAs) or in the form of proteins and in the processes/pathways mediated by them. However, and even for humans, there is still a tremendous lack of knowledge, that results from the complexity of the transcriptome, proteome and metabolome associated to the incredible heterogeneity of some diseases, such as cancer. Changes in the expression of many genes at the RNA and protein levels have been reported in feline mammary carcinomas. Although not on a human scale, progress has been made in respect to the feline mammary tumors’ expression profile and proteins’ repertoire. The expression profiling of the following genes transcripts or proteins has been conducted in FMCs over the last 17 years (Table 1), namely: BCL-2 [76], p53 [77], Cyclin A (apparently not overexpressed in feline mammary tumors [51]), Mst1R [48], ER [50,78], erbB-2 [2,41,63,78,79], COX-2 [80], Pten [75,81], Akt [82], mTOR [83], CXCR4 [84], catenins [85], cadherins [85,86], Twist-1 [49], claudins, CLDNs [87,88], Notch1 [89], PR, CK5/6 [78] or Ki-67 [78,90]. 

The *BCL-2* genes family encodes different proteins involved in apoptosis, amongst them, the pro-survival protein BCL-2 that has been used as a prognostic marker (e.g., [91]). Madewell and colleagues [76] examined the subcellular distribution of the BCL-2 protein in a collection of feline tumors immunohistochemically, and detected its presence in all mammary tumors. In human breast cancer, *BCL-2* was overexpressed in around 75% of the breast cancer samples analyzed by Dawson et al. [92], conferring the tumors the ability to evade apoptosis. The recent development of BH3 mimetic compounds that bind and neutralize BCL-2 have been developed [93] and have been used in hematological malignancies in early-phase therapies with a remarkable level of success [91]. The fact that this pro-survival protein is also overexpressed in human breast cancer has raised the interest in determining whether this new class of drug could aid in breast cancer treatment, however, much still needs to be done. As feline mammary tumors seem to mimic the human counterpart in overexpressing *BCL-2* [76], they seem to be suitable models to test this promising therapy.

In 2000, Nasir and colleagues [77] detected immunocytochemical staining of p53 (nuclear immunopositivity) in about one third of the feline mammary carcinomas tissues analyzed, indicating the presence of a mutant protein. p53 detected by immunostaining in neoplasia tissue sections (and at the nucleus) generally correlates with the presence of a mutation in the coding region of the gene and can be used as an indirect marker for p53 gene mutations (e.g., [94]). The high level of sequence homology between the *TP53* feline and human genes [95,96], in addition to the existence of mutant proteins, suggest that the mechanisms of tumorigenesis may be similar in humans and cats, indicating FMCs as a model for the study of this important gene in cancer.

DeMaria et al. [48] have identified the feline *MST1R* gene (member of the MET family of tyrosine kinase receptors) and studied its expression in feline mammary tumors. *MST1R* transcripts were detected by RT-PCR and the protein by immunohistochemical analysis in the majority of the feline mammary carcinomas examined. These authors concluded that the pattern of expression and localization of the MST1R protein in FMCs overlay with that of the homologous receptor in human breast cancer.

Recently, Soares et al. [78] analyzed the expression levels of different molecular markers; amongst these, the estrogen receptor (ER) in primary tumors and metastatic lesions. The majority of the metastases displayed loss of ER expression in comparison to the corresponding primary tumors, similarly to what has been reported in human breast cancer, where a considerable number of cases display discordance between primary and secondary sites [97,98,99]. Also, Cardazzo et al. [50] observed a decreased expression of ER in FMCs, mainly in some of the gene isoforms when compared to the normal gland tissue [50]. It seems that cats exhibiting ER-positive mammary tumors are associated with a better outcome, what also happens in human breast cancer (e.g., [78,100]). 

In 2005, De Maria et al. [2] identified the feline orthologue of the human *ERBB2* (displaying a similarity around 92%), an important prognostic marker and therapeutic target in human cancer. *ERBB2* is a proto-oncogene encoding the erbB-2 epithelial growth factor receptor protein [41]. *ERBB2* was found overexpressed in different mammary lesions either by RT-qPCR [2], Western blot [2] or immunohistochemistry [63,78,79]. Intriguingly, Santos and co-authors [41] seem to have obtained opposite results when analyzing cat mammary lesions by immunohistochemistry and RT-PCR, as they observed a general underexpression of the gene and of the protein level. Their results point to a subtype of carcinomas characterized by *ERBB2* RNA and erbB-2 protein underexpression. Also in some human breast cancers this has been observed [101,102]. These apparently contradictory data could be the result from the great heterogeneity of the disease, indicating that different gene expression statuses may occur and that the samples examined were not enough. However, it could also be due to the experimental conditions used in the different studies; namely, Santos et al. [41] seem to be the only group reporting the simultaneous use of antibodies for both the intra and extracellular domains of the protein. Additionally, there is variation in the cut-off values used by different authors and an absence of information concerning the normal respective mammary gland tissues in the different works. If indeed there are different subsets of feline mammary tumors displaying opposite *ERBB2* expression profiles, in parallel to what seems to happen in human breast cancer [101,102], then and once again, cat affirms itself as an extremely valuable model for either erbB-2 negative or positive [103] human breast cancer. 

Cyclooxygenase-2 (COX-2) is a protein involved in the biosynthesis of prostaglandins and plays a significant role in inflammation and tumorigenesis [104]. Sayasith et al. [80] analyzed, for the first time, the presence of this protein (immunohistochemically) in 40 feline mammary carcinomas and revealed that the majority of the tumors studied expressed COX-2 at variable levels. COX-2 is highly expressed in several human cancer stem cells, and it is thought to promote stem cell renewal, proliferation, and radioresistance (e.g., [105]), however, these processes are not yet fully understood but are thought to involve the down-regulation of *PDCD4* and *PTEN* [106]. *PTEN*, the phosphatase and tensin homolog, is a gatekeeper tumor suppressor gene [81]. *PTEN*-negative status occurred in 76% of the feline mammary carcinomas analyzed by Ressel et al. [81]. Furthermore, these authors found a significant correlation between loss of PTEN protein and lymphatic vessel invasion. *PTEN* down-regulation and *ERBB2* expression seems also to be caused by *AKT* activation (PI3K/AKT/PTEN pathway) in feline mammary tumors [82], as it occurs in human breast cancer (e.g., [107]). In 2013, Maniscalco [83] demonstrated that m-TOR is overexpressed and activated by phosphorylation via the *PI3K/AKT* signaling pathway in triple negative FMC, suggesting that these tumors might be suitable models to test innovative therapies against the human triple negative breast cancers, as mTOR is considered a potential target for anti-tumor therapies (e.g., [108]). It would be interesting to analyze COX-2, PTEN, Akt and m-TOR simultaneously as, in fact, it seems that the feline carcinoma once again behaves as the human cohort, and perhaps using this model will enable a better understanding of the exact interplay of these proteins and pathways, especially in triple negative tumors.

The chemokine/receptor pair SDF-1/CXCR4 has been involved in the regulation of metastization of neoplastic cells, including human breast cancer [109]. Ferrari et al. [84] demonstrated the expression of CXCR4 in feline mammary tumors, with malignant tumors displaying a higher level of expression. Furthermore, in the majority of the cases analyzed, metastatic cells displayed stronger immunoreactivity for CXCR4 than the corresponding primary tumors. Moreover, it seems that CXCR4 activation in primary cultures of FMCs increases the cellular proliferative rate. The tumorigenic effect exerted by CXCR4 in in vitro cultures from these tumor samples seems to be a good experimental model to investigate the biological and pharmacological role of this chemokinergic axis [84].

In 2012, Zappulli and co-authors [85] investigated immunohistochemically the adhesion glycoproteins E-cadherin and β-catenin in different mammary lesions and found a significant underexpression of both proteins in the malignant tumors and metastases. The E-cadherin/catenin complex is extremely important for the epithelial cell function, tissue integrity, and consequently cell-to-cell adhesion. In human breast cancer, a reduction in the expression of E-cadherin and its associated catenins was linked to invasiveness and metastization [110,111]. Decreased levels of the membrane E-cadherin, together with abnormalities in its location, were also described in feline mammary carcinomas, in comparison with benign tumors and hyperplastic or normal mammary tissue [85,112,113], where the preservation of E-cadherin was linked to inhibition of tumor cell invasiveness [85]. Takauji et al. [114], however, described variable expression of E-cadherin and two catenins in eight feline mammary tumor cell lines. In this way, it seems that other mechanisms must be causal for the acquisition of the infiltrative capability [115]. One of these mechanisms could be the switch from E-cadherin to N-cadherin described as a critical step in the malignant progression of neoplastic cells [116,117]. In fact, in 2014, Buendia and colleagues [86] analyzed a considerable collection of feline mammary tumors (21 adenomas and 139 carcinomas) and found a significant relationship between the expression of N-cadherin (immunohistochemically) and the two prognostic factors—the presence of regional metastasis and tumor grade [18,118], signs of malignancy. Furthermore, they observed a reduced expression of E-cadherin in the tumors expressing N-cadherin [86]. Figueira et al. [119] identified a direct relationship between the expression of another cadherin, P-cadherin and the aggressive biological behavior of feline mammary carcinomas. Together, these results suggest that N-cadherin and P-cadherin may be promising biomarkers of poor prognosis in FMCs. Moreover, Figueira et al. [119], suggest that the aberrant expression of P-cadherin is in fact a stronger marker for aggressiveness than the underexpression of E-cadherin.

Baptista et al. [49] detected low levels of expression (by RT-qPCR) of the oncogene *TWIST1* in feline mammary carcinomas when compared to benign mammary tumors. In human breast cancer, the analysis of *TWIST1* expression has shown to be variable. Low expression of Twist-1 protein and *TWIST1* mRNA was also detected in triple negative (estrogen receptor, progesterone receptor and HER-2 negative) breast invasive ductal carcinomas and was correlated with poor overall survival [120]. Gort et al. [121] found no significant differences in *TWIST1* mRNA levels between normal and malignant human breast tissue. On the contrary, Watanabe et al. [122] and Yang et al. [72] observed increased Twist-1 expression thought to be linked to mammary carcinogenesis. Moreover, an association of high Twist-1 protein expression was associated to a decrease in the survival time [123] and early systemic tumor relapse [124,125]. These contradictory results may be representative of the disease heterogeneity and its multifactorial pattern. Nevertheless, the observations of Baptista and colleagues [49], once again, indicate the use of cat as a model to analyze this oncogene behavior and role in tumorigenesis, most probably in triple negative breast cancer.

Claudins (CLDNs) are tight junction proteins involved in cell adhesion and polarity, paracellular permeability, proliferation and differentiation [87,88]. Several studies showed reduced expression of claudins in human (and canine mammary carcinomas) (e.g., [126,127,128]), suggesting that the decrease in these proteins contribute to mammary carcinogenesis, invasion and metastasis (e.g., [129,130]). Flores et al. [87,88] suggested that the evaluation of expression of CLDN-7 and CLDN-2 may be of prognostic and diagnostic value, specifically in a subgroup of aggressive FMCs that share some features with the recently characterized “claudin-low” subgroup of human breast cancer [131,132]. 

*NOTCH1* plays a role in many different human tumors including breast tumors [133]. It has been demonstrated that *NOTCH1* influences epidermal growth factor (*EGF*) receptor pathways [134], phosphate and tensin homologue (*PTEN*) [135,136], among others. Overexpression and aberrant localization of Notch intracellular domain (NICD) appears to be involved in the neoplastic transformation in feline mammary tissues [89]. Although further studies are required, the cytoplasmic localization of NICD suggests that an aberrant NOTCH1 pathway might occur in FMCs, differing from the established pathway and that could be involved in tumor initiation [89]. In human breast carcinomas, the role of NOTCH1 has been investigated (e.g., [137]). The active NOTCH1 signaling pathway is thought to be a major survival pathway used by cancer cells to overcome different therapeutics. NOTCH1 is overexpressed in tumors unresponsive to radiotherapy [138] or molecular targeted therapy [139]. Osipo et al. [140] revealed that in Trastuzumab-treated, HER-2-positive or Tamoxifen-treated estrogen receptor (ER)-positive human mammary tumors, the NOTCH1 pathway that is initially inactivated is turned on after a long period of chemotherapy. Much is still to be done in this respect, as the tumor cells have shown the ability to find alternative escape mechanisms that confer the capacity to overcome therapeutic obstacles, acquiring resistance overtime. The follow-up studies of the cat animal model or of in vitro cell lines could be a good strategy to track tumor progression and its genomic changes over time and to develop targeted therapies.

Other prognostic biomarkers have been used in feline mammary tumors confirming the similarities between FMC and HBC, such as metallothioneins [141], VEGF [142], PR, CK5/6 [78] or Ki-67 [78,90]. Soares et al. [78] found a great heterogeneity of these protein levels between primary tumors and their respective metastases, where estrogen and progesterone receptors usually underexpressed in metastases. Regarding Ki-67, metastases showed the higher index for this marker when compared to their primary tumors [90]. Ki-67 is a protein found only in proliferative cells and expressed in all the cell cycle phases (except G0), although in a precise level fashion for each phase. This makes Ki-67 a useful and reliable proliferation biomarker in human breast cancer [143,144] that can also be used as a prognostic biomarker in feline mammary carcinoma, according to the recent studies by Soares et al. [89], due to the similarity that these biomarkers display in both human and cat mammary tumors. 

The data collected here highlights the prognostic and diagnostic importance of the molecular phenotype assessment (using diverse technologies as immunocyto(histo)chemistry, RT-qPCR, etc.) in feline mammary carcinomas, as in human breast cancer. The variable protein expression most of the times observed between local tumor and metastases justifies a precise knowledge of the pathways involved. Altogether, the data collected clearly demonstrates that FMC can be used as a model to study human breast cancer. A note has to be made on the technical challenges of using not only the cat, but many other model animals that rely on the almost absence of species-specific experimental tools, such as monoclonal antibodies, nucleic acid probes, DNA libraries, reagents, among others (e.g., [145]), implying a large amount of time and expenses to optimize the methodologies.

## 5. Cat Mammary Tumor Cell Lines—Suitable Cell Tools, but Still with Limited Availability

Cat mammary tumor cell lines are extremely valuable as in vitro models for cancer research and may have a great clinical relevance once they are characterized in detail. In fact, its characterization is fundamental to provide important insights about the complexity of the aetiology of cancer and the biological mechanisms involved in this pathology [146], allowing a thinker choice of the cell line in accordance with the research to be carried out. For instance, cell lines can provide adequate cellular models to: analyze the origin of cancers by the presence of initiating cancer cells or cancer stem cells [147,148]; analyze carcinogenesis through the phenotype and genotype evolutionary study in order to understand the cancer progression until metastasis [149,150]; develop and test drugs as a first approach to targeting therapies [147,148,149]; screen RNAi (RNA interference) libraries and other small molecules as a way to study interacting pathways in the initiation and survival of tumors [147,148,149,150]; amongst many other examples, which can be reviewed in the literature (e.g., [150]). 

In spite of the crucial role of cancer cell lines as tumor cell models, a controversial issue emerges if they may not fully represent the original tumor [150,151,152]. Even though, there is a high genomic similarity between both the original tumor and their derived cancer cell lines [149,150,153,154]. Also, it is worth mentioning that patient tumor cells implanted as orthotopic tumor grafts are an emergent alternative model to the in vitro conditions, as those models more accurately reflect the three-dimensional tumor microenvironment [155]. All the other models used to study carcinogenesis display several drawbacks. Just to give one example, fresh tumor samples or tumor samples embedded in paraffin are limited in their amount, only revealing a specific evolutionary *momento* of the tumor [148], and may not be archetypal of the original tumor due to its natural tumor heterogeneity [146]. It is clear that animal models exhibiting spontaneous tumors (as the domestic cat) have a huge advantage in providing a tumor historical sequence and are utmost for testing new therapeutics in vivo (once they are inserted in the genuine microenvironment) [148]. Still, the cell lines derived from the same species of the animal model should be the first line of research due to obvious ethical concerns associated with animal testing, which further emphasizes the importance of using cat tumor cell lines. Unfortunately, the number of cat tumor cell lines, and specifically derived from the mammary gland, are very scarce. The first report describing a long term feline mammary tumor cell line named JM was published in 1985 by Norval et al. [156], now available as CAT-MT in the European Collection of Authenticated Cell Cultures—ECACC. Minke et al. [20] reported two distinct epithelial cell lines derived from a single feline mammary carcinoma with different tumorigenic potential. Muleya et al. [157] presented a tumor feline cell line named by FRM and Uyama et al. [158] published the establishment and characterization of eight feline mammary adenocarcinoma cell lines. Finally, this year Borges and colleagues [24] published a new feline mammary tumor cell line, designated FkMTp, which has the particularity of being cryopreserved every six passages, allowing the possibility to access almost any specific *momento* of the in vitro tumor progression; in fact, it is a collection of around 23 “cell lines” derived from the same piece of tumor.

In resume, the availability of a larger number of cell lines is of great interest, enabling the domestic cat to be suitably used as a cell and animal model.

## 6. Conclusions

The most appropriate models provide essential conceptual tools for basic and clinical research, granting a better understanding of breast cancer biology, and consequently allowing a more accurate stratification of patients for targeted therapies. This knowledge will be critical not only for the development of novel anticancer approaches, but also—and perhaps more importantly—to allow a more efficient use of the currently available drugs [59]. The cat seems to have all the ingredients to become a golden alternative cancer model, and undoubtedly a superior cancer genetics model in comparison to others commonly used. However, we still need to boost research in cat cancer genetics to deliver the most suitable and usable models. 

## Figures and Tables

**Table 1:** This table summarizes the critical cancer genes already analyzed in feline mammary carcinomas.

Gene	Type/Name	DNA Mutations	Expression Profile	Protein	References
*TP53*	Suppressor gene	Missense mutations and deletions	Mutant proteins Nuclear immunopositivity	p53	Mayr et al. 2000 [60], Mayr et al. 1995 [61], Mayr et al. 1998 [62], Nasir et al. 2000 [77]
*ERRB2*	Growth factor receptor tyrosine kinase 2	Sequence variants/haplotypes non-synonymous mutations	Underexpression Overexpression	erbB-2	Santos et al. 2012 [4], Santos et al. 2013 [41], De Maria et al. 2005 [2], Órdas et al. 2007 [79], Soares et al. 2013 [63], Soares et al. 2016 [78]
*MST1R*	Tyrosine kinase receptor	Point mutations	Overexpression	MST1R	De Maria et al. 2002 [48]
*ER*	Estrogen receptor	Sequence/isoform variants	Exon deleted splicing variants Underexpression in metastases when compared to the primary tumors	ER	Cardazzo et al. 2005 [50], Soares et al. 2016 [78]
*TWIST1*	Oncogene	Intronic germline sequence variants	Underexpression Overexpression	Twist-1	Baptista et al. 2012 [49], Yang et al. 2004 [72], Watanabe et al. 2004 [122]
*BCL-2*	Protein Phosphatase 1 Blocks the apoptotic death	No information	Overexpression	pro-survival protein BCL-2	Madewell et al. 1999 [76]
*CCNA2*	Cyclin A Regulator of CDK kinases	No information	Regular expression	Cyclin A	Murakami et al. 2000 [51]
*COX2*	Cytochrome C Oxidase Assembly Factor	No information	Variable expression	COX-2	Sayasith et al. 2009 [80]
*PTEN*	Suppressor gene Phosphatase and Tensin homolog	No information	Underexpression	PTEN	Ressel et al. 2009 [81]
*AKT*	Serine-threonine protein kinase	No information	Overexpression	Akt	Maniscalco et al. 2012 [82]
*mTOR*	Mechanistic Target of Rapamycin (Serine/Threonine Kinase)	No information	Overexpression and activation by phosphorylation via the *PI3K/AKT* signaling pathway in triple negative FMCs	mTOR	Maniscalco et al. 2013 [83]
*CXCR4*	Chemokine (CXC Motif) Receptor 4	No information	Overexpression	CXCR4	Ferrari et al. 2012 [84]
*CTNNs*	Cadherin-Associated Protein	No information	Underespression	Catenins	Zappulli et al. 2012 [85]
*CDH1* *CDH2*	Adhesion proteins	No information	Underexpression and abnormal cellular location	E-cadherin N-cadherin	Zappulli et al. 2012 [85], Buendia et al. 2014 [86]
*CDH3*	Adhesion proteins	No information	Overexpression	P-cadherin	Figueira et al. 2014 [119]
*CLDN2* *CLDN7*	Tight junctions membrane proteins	No information	Underexpression	Claudins 2 and 7	Flores et al. 2014a, b [87,88]
*NOTCH1*	Transmembrane protein	No information	Overexpression and aberrant location	NOTCH-1	Ressel et al. 2014 [89]
*PR*	Progesterone Receptor	No information	Underexpression in metastases	PR	Soares et al. 2016 [78]
*KRT 5/6*	Cytokeratin Intermediate filament protein	No information	Variable expression	CK5/6	Soares et al. 2016 [78]
*MKI67*	Nuclear protein associated to cellular proliferation	No information	Overexpression in metastases	Ki-67	Soares et al. 2016 [90]
